# 4,12-Diselena-5,6,13,14-tetra­aza­tri­cyclo­[9.3.0.0^3,7^]tetra­deca-1(11),3(7),5,13-tetra­ene

**DOI:** 10.1107/S2414314625003244

**Published:** 2025-05-02

**Authors:** Dieter Schollmeyer, Heiner Detert

**Affiliations:** a University of Mainz, Department of Chemistry, Duesbergweg 10-14, 55099 Mainz, Germany; Katholieke Universiteit Leuven, Belgium

**Keywords:** crystal structure, heterocycle, selenium, medium-sized ring

## Abstract

In the title com­pound, C_8_H_8_N_4_Se_2_, two almost planar 1,2,3-selena­diazo­les are annulated to a cyclo­octa-1,4-diene with a boat–chair conformation, giving the mol­ecule a butterfly shape.

## Abstract 2

Two almost planar 1,2,3-selena­diazo­les are annulated to a cyclo­octa-1,4-diene with a boat–chair conformation, giving the mol­ecule a butterfly shape.

## Graphical abstract

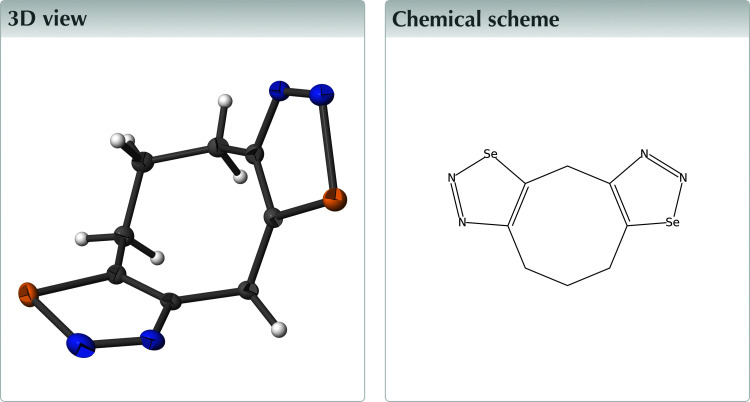

## Structure description

**Figure 1:**
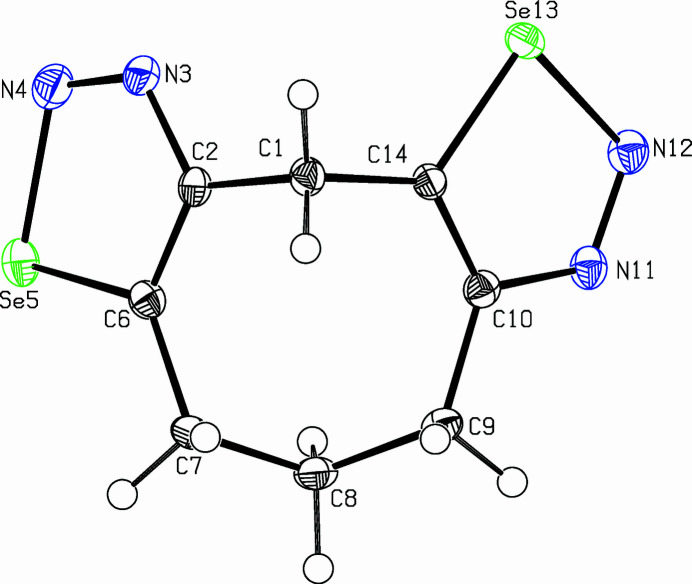
View of the title com­pound, with displacement ellipsoids drawn at the 50% probability level.

**Figure 2:**
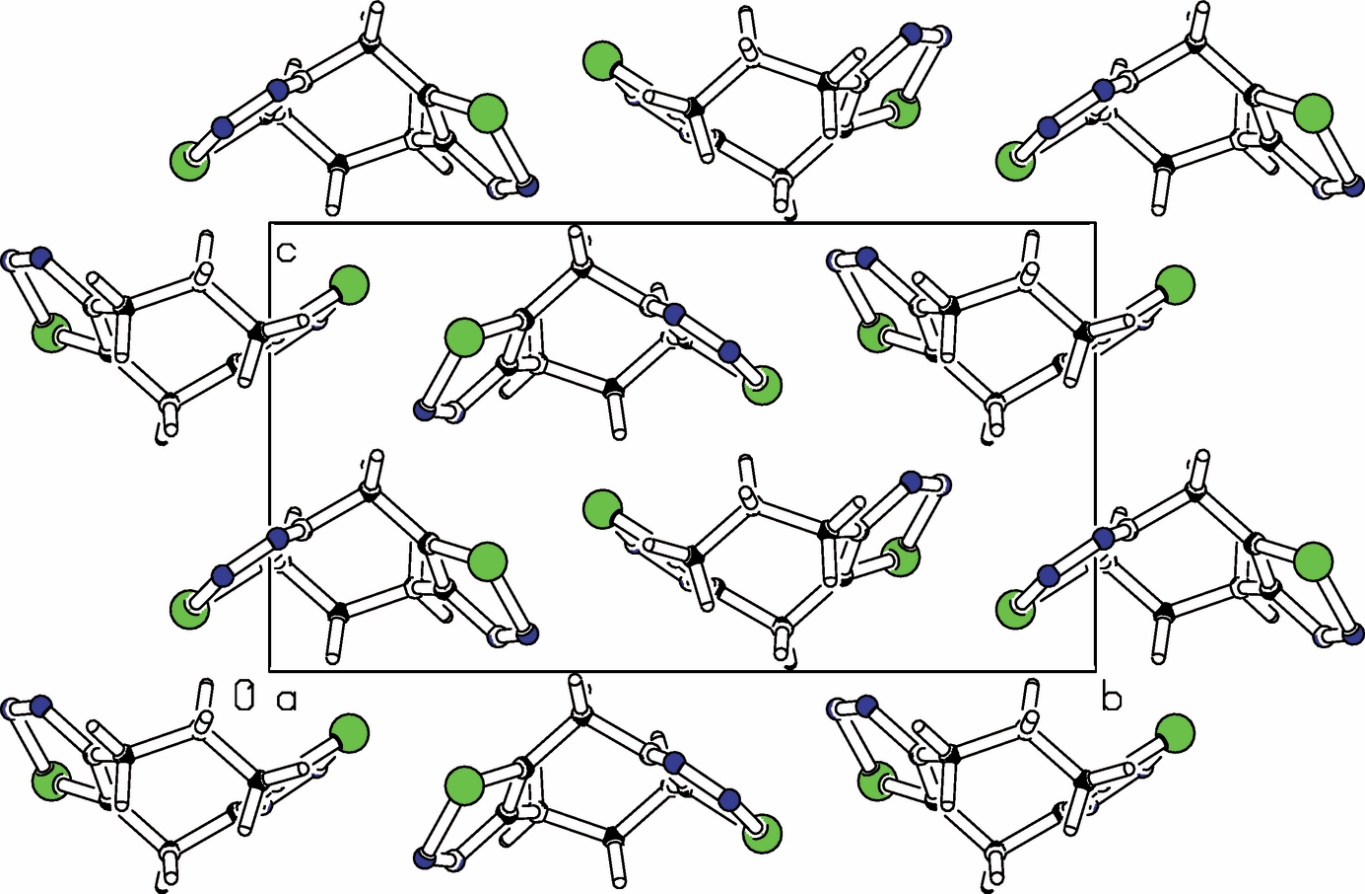
Part of the packing diagram, viewed along the *a*-axis direction.

The title com­pound, C_8_H_8_N_4_Se_2_, was prepared as part of a project focusing on medium-sized cyclo­alkynes with additional sterically demanding groups (Bissinger *et al.*, 1988 ▸; Detert *et al.*, 1994 ▸; Detert & Meier, 1997 ▸). Bis-1,2,3-selana­diazo­les are important sources for medium-sized cyclo­alkadiynes (Gleiter *et al.*, 1988 ▸) and the structure of an isomer of the title com­pound has recently been reported (Detert & Schollmeyer, 2020 ▸). The tricyclic mol­ecule adopts a butterfly-like shape with a boat–chair conformation of the eight-membered ring and two 1,2,3-selena­diazo­le rings are fused to the central ring (Fig. 1 ▸). Selena­diazole ring 1 (C2—N3—N4—Se5—C6) is planar within 0.003 (3) Å and selena­diazole ring 2 (C10—N11—N12—Se13—C14) within 0.008 (3) Å. While the connecting C1 atom lies above the plane of both selena­diazo­le rings [selena­diazole 1: 0.130 (3) Å; selena­diazole 2: 0.118 (3) Å], the adjacent C atoms of the propyl­ene tether are either above these planes [C7: 0.037 (3) Å] or below [C9: −0.134 (3) Å]. The planes of the selena­diazo­le rings subtend a dihedral angle of 79.64 (13)°. Strain in the medium-sized ring is reflected in distortion of the bond angles on C7 [113.8 (3)°], C8 [115.8 (3)°] and C9 [118.4 (3)°], whereas a bond angle of 108.3 (2)° for C2—C1—C14 is close to the perfect tetra­hedral angle. The packing diagram of the title com­pound is shown in Fig. 2 ▸.

## Synthesis and crystallization

The title com­pound was prepared from cyclo­octane-1,4-diol *via* Jones oxidation, formation of the semicarbazone and reaction with selenous acid in a 4.4% overall yield [m.p. 398–400 K (decom­position)]. Crystals were grown by slow evaporation of a solution in chloro­form–propan-2-ol. ^1^H NMR (400 MHz, CDCl_3_): δ 5.01 (*s*, 2H, H_2_C-2), 3.25 and 3.10 (each: *t*, 2H, *J* = 6.6 Hz, H_2_C-8,10), 1.95 (pseudo-*q*, 2H, H_2_C-9). ^13^C NMR (100 MHz, CDCl_3_): δ 159.4, 158.9, 156.4, 156.1 (C-1, 2, 7, 11); 26.7 (C-2), 26.4 (C-9), 25.7, 24.7 (C-8, 10) 41.5, 29.4, 28.5, 27.4, 25.5, 22.5, 20.0. ^77^Se NMR (73 MHz, CDCl_3_, SeO_2_/D_2_O as reference): δ 241.3, 240.7; UV (EtOH): λ (logɛ): 222 (4.09), 287 nm (3.45), MS (FD): 320 (*M*^+^, Se_2_-isotope pattern), 292 (*M*^+^—N_2_, Se_2_-isotope pattern), 264 (*M*^+^—N_2_, Se_2_-isotope pattern).

## Refinement

Crystal data, data collection and structure refinement details are summarized in Table 1 ▸. H atoms attached to C atoms were placed at calculated positions and were refined in the riding-model approximation, with C—H = 0.99 Å and *U*_iso_(H) = 1.2 *U*_eq_(C).

## Supplementary Material

Crystal structure: contains datablock(s) I, global. DOI: 10.1107/S2414314625003244/vm4066sup1.cif

Structure factors: contains datablock(s) I. DOI: 10.1107/S2414314625003244/vm4066Isup2.hkl

Supporting information file. DOI: 10.1107/S2414314625003244/vm4066Isup3.cml

CCDC reference: 2442669

Additional supporting information:  crystallographic information; 3D view; checkCIF report

## Figures and Tables

**Table 1:** Experimental details

Crystal data	
Chemical formula	C_8_H_8_N_4_Se_2_
*M*_r_	318.10
Crystal system, space group	Monoclinic, *P*2_1_/*n*
Temperature (K)	120
*a*, *b*, *c* (Å)	7.2217 (4), 15.6664 (8), 8.4925 (5)
β (°)	90.110 (4)
*V* (Å^3^)	960.82 (9)
*Z*	4
Radiation type	Mo *K*α
μ (mm^−1^)	7.66
Crystal size (mm)	0.42 × 0.31 × 0.21

Data collection	
Diffractometer	STOE IPDS 2T
Absorption correction	Integration
*T*_min_, *T*_max_	0.107, 0.251
No. of measured, independent and observed [*I* > 2σ(*I*)] reflections	5404, 2296, 2066
*R*_int_	0.022
(sin θ/λ)_max_ (Å^−1^)	0.660

Refinement	
*R*[*F*^2^ > 2σ(*F*^2^)], *wR*(*F*^2^), *S*	0.029, 0.071, 1.17
No. of reflections	2296
No. of parameters	127
H-atom treatment	H-atom parameters constrained
Δρ_max_, Δρ_min_ (e Å^−3^)	0.48, −0.44

Computer programs: *WinXpose* in *X-AREA* (Stoe & Cie, 2020 ▸), *Recipe* in *X-AREA* (Stoe & Cie, 2020 ▸), *Integrate* in *X-AREA* (Stoe & Cie, 2020 ▸), *SHELXT2014* (Sheldrick, 2015*a* ▸), *SHELXL2019* (Sheldrick, 2015*b* ▸) and *PLATON* (Spek, 2020 ▸).

## full crystallographic data

### 4,12-Diselena-5,6,13,14-tetraazatricyclo[9.3.0.03,7]tetradeca-1(11),3(7),5,13-tetraene Crystal data

**Table:**

C_8_H_8_N_4_Se_2_	*F*(000) = 608
*M**_r_* = 318.10	*D*_x_ = 2.199 Mg m^−^^3^
Monoclinic, *P*2_1_/*n*	Mo *K*α radiation, λ = 0.71073 Å
*a* = 7.2217 (4) Å	Cell parameters from 11184 reflections
*b* = 15.6664 (8) Å	θ = 2.7–28.3°
*c* = 8.4925 (5) Å	µ = 7.66 mm^−^^1^
β = 90.110 (4)°	*T* = 120 K
*V* = 960.82 (9) Å^3^	Block, colorless
*Z* = 4	0.42 × 0.31 × 0.21 mm

### 4,12-Diselena-5,6,13,14-tetraazatricyclo[9.3.0.03,7]tetradeca-1(11),3(7),5,13-tetraene Data collection

**Table:**

STOE IPDS 2T diffractometer	2296 independent reflections
Radiation source: sealed X-ray tube, 12x0.4mm long-fine focus	2066 reflections with *I* > 2σ(*I*)
Detector resolution: 6.67 pixels mm^-1^	*R*_int_ = 0.022
rotation method, ω scans	θ_max_ = 28.0°, θ_min_ = 2.7°
Absorption correction: integration	*h* = −9→8
*T*_min_ = 0.107, *T*_max_ = 0.251	*k* = −20→20
5404 measured reflections	*l* = −11→9

### 4,12-Diselena-5,6,13,14-tetraazatricyclo[9.3.0.03,7]tetradeca-1(11),3(7),5,13-tetraene Refinement

**Table:**

Refinement on *F*^2^	Primary atom site location: dual
Least-squares matrix: full	Hydrogen site location: inferred from neighbouring sites
*R*[*F*^2^ > 2σ(*F*^2^)] = 0.029	H-atom parameters constrained
*wR*(*F*^2^) = 0.071	*w* = 1/[σ^2^(*F*_o_^2^) + (0.0269*P*)^2^ + 2.435*P*] where *P* = (*F*_o_^2^ + 2*F*_c_^2^)/3
*S* = 1.17	(Δ/σ)_max_ = 0.001
2296 reflections	Δρ_max_ = 0.48 e Å^−^^3^
127 parameters	Δρ_min_ = −0.44 e Å^−^^3^
0 restraints	

### 4,12-Diselena-5,6,13,14-tetraazatricyclo[9.3.0.03,7]tetradeca-1(11),3(7),5,13-tetraene Special details

**Table:**

Geometry. All esds (except the esd in the dihedral angle between two l.s. planes) are estimated using the full covariance matrix. The cell esds are taken into account individually in the estimation of esds in distances, angles and torsion angles; correlations between esds in cell parameters are only used when they are defined by crystal symmetry. An approximate (isotropic) treatment of cell esds is used for estimating esds involving l.s. planes.

### 4,12-Diselena-5,6,13,14-tetraazatricyclo[9.3.0.03,7]tetradeca-1(11),3(7),5,13-tetraene Fractional atomic coordinates and isotropic or equivalent isotropic displacement parameters (Å^2^)

**Table:**

	*x*	*y*	*z*	*U*_iso_*/*U*_eq_	
C1	0.4215 (4)	0.37887 (19)	0.9033 (4)	0.0160 (6)	
H1A	0.302532	0.380853	0.960943	0.019*	
H1B	0.522437	0.369867	0.980596	0.019*	
C2	0.4515 (4)	0.46134 (19)	0.8159 (3)	0.0152 (6)	
N3	0.6299 (4)	0.49038 (17)	0.7962 (3)	0.0178 (5)	
N4	0.6528 (4)	0.55671 (18)	0.7130 (3)	0.0221 (6)	
Se5	0.42162 (5)	0.59667 (2)	0.63795 (4)	0.02054 (10)	
C6	0.3146 (4)	0.5069 (2)	0.7443 (4)	0.0157 (6)	
C7	0.1118 (4)	0.4872 (2)	0.7445 (4)	0.0180 (6)	
H7A	0.042450	0.539719	0.717557	0.022*	
H7B	0.074926	0.469928	0.852125	0.022*	
C8	0.0561 (4)	0.4161 (2)	0.6286 (4)	0.0185 (6)	
H8A	−0.076361	0.423468	0.601538	0.022*	
H8B	0.128039	0.423644	0.530446	0.022*	
C9	0.0848 (4)	0.3240 (2)	0.6872 (4)	0.0171 (6)	
H9A	0.039233	0.321004	0.796966	0.020*	
H9B	0.004683	0.286199	0.623171	0.020*	
C10	0.2770 (4)	0.2876 (2)	0.6847 (3)	0.0153 (6)	
N11	0.3132 (4)	0.22323 (17)	0.5779 (3)	0.0175 (5)	
N12	0.4723 (4)	0.18783 (17)	0.5841 (3)	0.0194 (5)	
Se13	0.61533 (4)	0.23617 (2)	0.74477 (4)	0.01775 (9)	
C14	0.4189 (4)	0.30689 (19)	0.7851 (3)	0.0141 (6)	

### 4,12-Diselena-5,6,13,14-tetraazatricyclo[9.3.0.03,7]tetradeca-1(11),3(7),5,13-tetraene Atomic displacement parameters (Å^2^)

**Table:**

	*U*^11^	*U*^22^	*U*^33^	*U*^12^	*U*^13^	*U*^23^
C1	0.0178 (14)	0.0145 (14)	0.0158 (13)	0.0004 (11)	−0.0014 (11)	−0.0020 (11)
C2	0.0175 (14)	0.0135 (13)	0.0147 (13)	−0.0003 (11)	−0.0004 (11)	−0.0039 (11)
N3	0.0174 (13)	0.0166 (12)	0.0195 (12)	−0.0013 (10)	−0.0014 (10)	−0.0040 (10)
N4	0.0211 (14)	0.0207 (14)	0.0246 (14)	−0.0032 (11)	0.0018 (11)	−0.0046 (11)
Se5	0.02600 (18)	0.01483 (16)	0.02079 (16)	−0.00047 (12)	0.00078 (13)	0.00212 (11)
C6	0.0169 (14)	0.0152 (14)	0.0151 (13)	0.0011 (11)	0.0004 (11)	−0.0006 (11)
C7	0.0142 (14)	0.0180 (15)	0.0217 (15)	0.0037 (11)	−0.0019 (12)	−0.0011 (12)
C8	0.0120 (14)	0.0218 (16)	0.0217 (15)	0.0006 (11)	−0.0015 (12)	−0.0011 (12)
C9	0.0132 (14)	0.0198 (15)	0.0182 (14)	−0.0017 (11)	0.0005 (11)	−0.0005 (12)
C10	0.0157 (14)	0.0150 (13)	0.0152 (13)	−0.0011 (11)	−0.0002 (11)	0.0016 (11)
N11	0.0217 (13)	0.0156 (12)	0.0153 (12)	−0.0004 (10)	−0.0012 (10)	−0.0009 (10)
N12	0.0224 (14)	0.0193 (13)	0.0165 (12)	−0.0003 (11)	−0.0011 (10)	−0.0020 (10)
Se13	0.01585 (16)	0.01742 (16)	0.01997 (16)	0.00329 (11)	−0.00227 (11)	−0.00193 (11)
C14	0.0143 (14)	0.0125 (13)	0.0155 (13)	0.0007 (11)	0.0007 (11)	−0.0002 (10)

### 4,12-Diselena-5,6,13,14-tetraazatricyclo[9.3.0.03,7]tetradeca-1(11),3(7),5,13-tetraene Geometric parameters (Å, º)

**Table:**

C1—C2	1.506 (4)	C7—H7B	0.9900
C1—C14	1.510 (4)	C8—C9	1.540 (4)
C1—H1A	0.9900	C8—H8A	0.9900
C1—H1B	0.9900	C8—H8B	0.9900
C2—C6	1.362 (4)	C9—C10	1.501 (4)
C2—N3	1.377 (4)	C9—H9A	0.9900
N3—N4	1.268 (4)	C9—H9B	0.9900
N4—Se5	1.892 (3)	C10—C14	1.366 (4)
Se5—C6	1.842 (3)	C10—N11	1.382 (4)
C6—C7	1.497 (4)	N11—N12	1.277 (4)
C7—C8	1.540 (4)	N12—Se13	1.870 (3)
C7—H7A	0.9900	Se13—C14	1.833 (3)
			
C2—C1—C14	108.3 (2)	C9—C8—C7	115.8 (3)
C2—C1—H1A	110.0	C9—C8—H8A	108.3
C14—C1—H1A	110.0	C7—C8—H8A	108.3
C2—C1—H1B	110.0	C9—C8—H8B	108.3
C14—C1—H1B	110.0	C7—C8—H8B	108.3
H1A—C1—H1B	108.4	H8A—C8—H8B	107.4
C6—C2—N3	116.8 (3)	C10—C9—C8	118.4 (3)
C6—C2—C1	124.4 (3)	C10—C9—H9A	107.7
N3—C2—C1	118.6 (3)	C8—C9—H9A	107.7
N4—N3—C2	117.5 (3)	C10—C9—H9B	107.7
N3—N4—Se5	110.0 (2)	C8—C9—H9B	107.7
C6—Se5—N4	87.31 (13)	H9A—C9—H9B	107.1
C2—C6—C7	126.9 (3)	C14—C10—N11	115.4 (3)
C2—C6—Se5	108.3 (2)	C14—C10—C9	126.9 (3)
C7—C6—Se5	124.7 (2)	N11—C10—C9	117.6 (3)
C6—C7—C8	113.8 (3)	N12—N11—C10	117.5 (3)
C6—C7—H7A	108.8	N11—N12—Se13	110.5 (2)
C8—C7—H7A	108.8	C14—Se13—N12	87.40 (13)
C6—C7—H7B	108.8	C10—C14—C1	126.1 (3)
C8—C7—H7B	108.8	C10—C14—Se13	109.3 (2)
H7A—C7—H7B	107.7	C1—C14—Se13	124.6 (2)
			
C14—C1—C2—C6	83.3 (4)	C7—C8—C9—C10	−78.1 (4)
C14—C1—C2—N3	−91.8 (3)	C8—C9—C10—C14	74.3 (4)
C6—C2—N3—N4	−0.2 (4)	C8—C9—C10—N11	−110.8 (3)
C1—C2—N3—N4	175.2 (3)	C14—C10—N11—N12	0.7 (4)
C2—N3—N4—Se5	−0.1 (3)	C9—C10—N11—N12	−174.7 (3)
N3—N4—Se5—C6	0.3 (2)	C10—N11—N12—Se13	0.2 (3)
N3—C2—C6—C7	178.3 (3)	N11—N12—Se13—C14	−0.8 (2)
C1—C2—C6—C7	3.1 (5)	N11—C10—C14—C1	174.7 (3)
N3—C2—C6—Se5	0.5 (3)	C9—C10—C14—C1	−10.4 (5)
C1—C2—C6—Se5	−174.7 (2)	N11—C10—C14—Se13	−1.3 (3)
N4—Se5—C6—C2	−0.4 (2)	C9—C10—C14—Se13	173.7 (2)
N4—Se5—C6—C7	−178.3 (3)	C2—C1—C14—C10	−75.3 (4)
C2—C6—C7—C8	−78.4 (4)	C2—C1—C14—Se13	100.0 (3)
Se5—C6—C7—C8	99.1 (3)	N12—Se13—C14—C10	1.1 (2)
C6—C7—C8—C9	82.1 (3)	N12—Se13—C14—C1	−174.9 (3)
